# Recent Advances in Bioanalytical Methods for Quantification and Pharmacokinetic Analyses of Antibody–Drug Conjugates

**DOI:** 10.1208/s12248-025-01115-9

**Published:** 2025-09-04

**Authors:** R. M. Naseer Khan, Yi Zeng, Abdul‑Azeez A. Lanihun, Oluwatobi T. Arisa, Jessica L. Horner, William D. Figg

**Affiliations:** 1 Clinical Pharmacology Laboratory, Clinical Center, National Institutes of Health, 9000 Rockville Pike, Building 10, Room 5A03, Bethesda, Maryland 20892, USA; 2 Clinical Pharmacology Program, Office of the Clinical Director, Center for Cancer Research, National Cancer Institute, Bethesda, Maryland, USA

**Keywords:** analytical chemistry, antibody–drug conjugate, bioanalytical, cancer, clinical Pharmacology, ECLIA, ELISA, FT-ICR, LC–MS/MS, pharmacokinetic

## Abstract

Antibody–drug conjugates (ADCs) represent a rapidly expanding class of therapeutics, uniquely combining the specificity of monoclonal antibodies with the potency of cytotoxic small-molecule payloads. Due to their inherent structural complexity and heterogeneous composition, accurate characterization and quantification of ADCs pose significant bioanalytical challenges. This review discusses recent advancements in bioanalytical methodologies, including ligand binding assays (LBAs), liquid chromatography-tandem mass spectrometry (LC–MS/MS)-based approaches, and emerging hybrid LBA-LC–MS/MS platforms. In addition, this review will discuss pharmacokinetic (PK) modeling approaches essential to ADC development, ranging from population PK models to mechanistic frameworks, including physiologically based pharmacokinetic (PBPK) and quantitative systems pharmacology (QSP) models. These modeling strategies allow detailed characterization of ADC absorption, distribution, metabolism, and elimination processes while also accounting for complexities introduced by payload deconjugation and drug-to-antibody ratio variability. By integrating robust bioanalytical methods with advanced modeling techniques, this review provides researchers with essential insights to enhance ADC characterization, inform experimental design, and ultimately facilitate the development of safer, more effective therapeutic candidates.

## Introduction

Antibody–drug conjugates (ADCs) are a fast-emerging class of chemotherapeutics, with over a dozen approved by the U.S. Food and Drug Administration (FDA) to date (see Table I). While some, such as Lumoxiti and Blenrep, have since been withdrawn from the market, more than 100 ADCs remain in clinical trials for various cancer treatments (1). In 2000, gemtuzumab ozogamicin became the first ADC to receive accelerated FDA approval for the treatment of acute myeloid leukemia (AML) (2). It was initially withdrawn due to safety concerns, but later reapproved following adjustments to its dosing regimen that improved its safety and efficacy profile. Since then, multiple ADCs have been approved and have become essential for the treatment of various cancers, including hematologic malignancies, as well as urothelial, lung, and breast cancers. A recent example is datopotamab deruxtecan, approved for the treatment of unresectable or metastatic, hormone receptor-positive, human epidermal growth factor receptor 2 (HER2)-negative breast cancer (3).

**Fig. 1:**
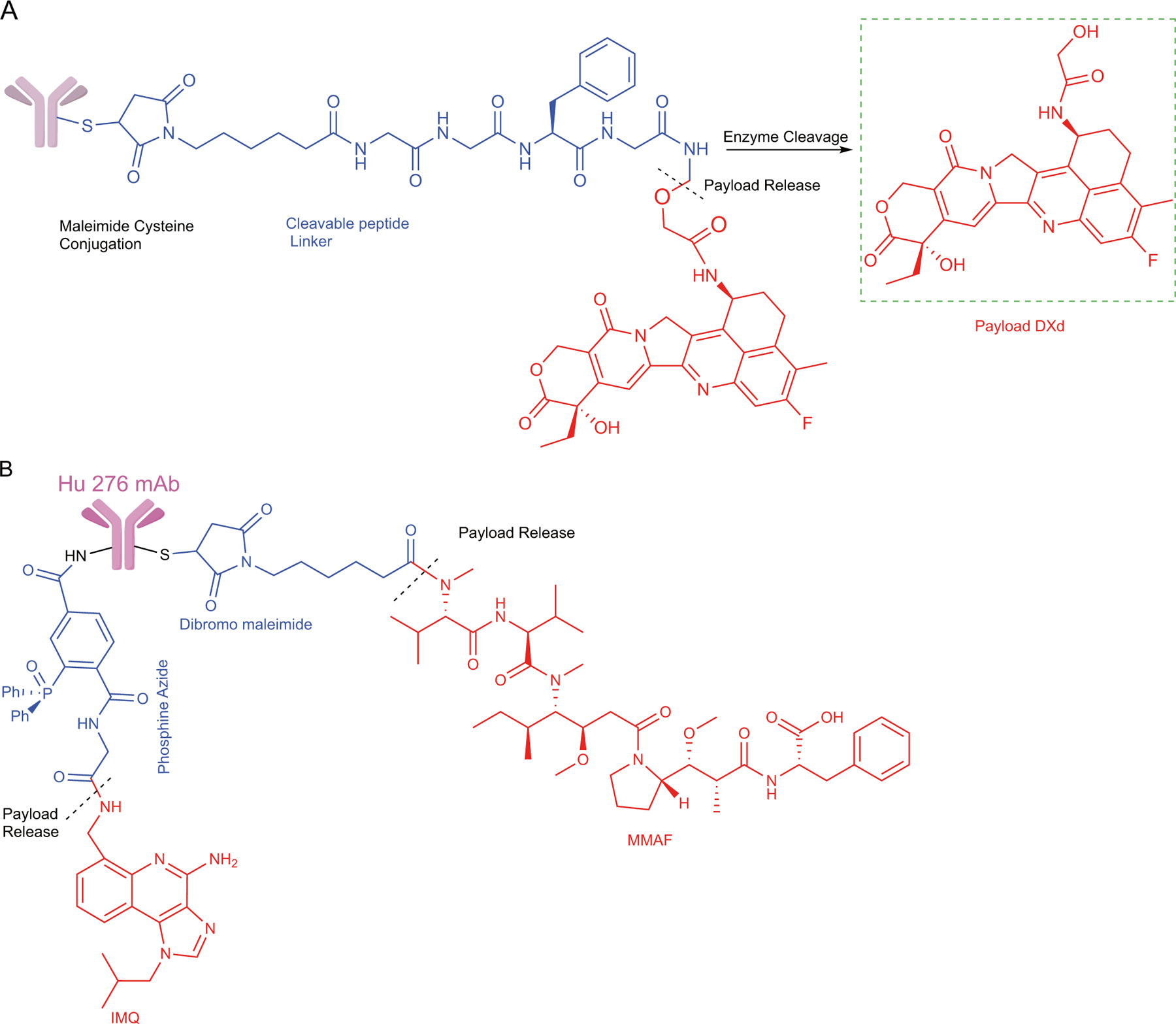
Schematic depiction of various conjugation strategies utilized during the design phase of single and dual-payload antibody–drug conjugates (ADCs). **a** The structure of the recently FDA-approved datopotomab deruxtecan, which comprises a topoisomerase I inhibitor (DXd payload), covalently attached to the antibody via a tetrapeptide-based cleavable linker. The cytotoxic payload is conjugated through maleimide-cysteine linkage. **b** The structure of the dual-payload ADC, CD276 monoclonal antibody (mAb) conjugated to monomethyl auristatin F (MMAF) and imiquimod (IMQ), linked through either cysteine or lysine residues using two distinct linkers. Payloads are shown in color red; cleavable peptide linkers are shown in color blue and conjugation site is shown in color black. Adapted from references 36 and 59 and created using ChemDraw

ADCs are designed to selectively deliver potent cytotoxic agents to cancer cells and consist of three key components: a cytotoxic payload, an antibody, and a linker that covalently attaches the payload to amino acids on the antibody (4). ADCs can incorporate multiple linkers, increasing the concentration of payloads delivered. These are typically conjugated to the antibody via lysine residues or reduced interchain disulfide bonds, resulting in a heterogeneous mixture of ADCs with variable drug–antibody ratios (DAR 0–8) (see Fig. 1) (5–7). Notably, deconjugation of the cytotoxic payload, occurring either enzymatically or chemically can take place in systemic circulation, resulting in a gradual decrease in the average DAR over time. While linker stability primarily impacts ADC safety and efficacy by increasing the systemic levels of the cytotoxic payload, systemic deconjugation can complicate bioanalytical assays by increasing analyte heterogeneity and can pose additional challenges for accurate pharmacokinetic (PK) analysis (8, 9). Thus, during the design stage of an ADC, selecting a linker with substantial *in vivo* stability is a critical parameter (10). Both cleavable and non-cleavable linkers have been used in approved ADCs, and there has been continuous progress in linker technology to prevent premature release of cytotoxic drugs in non-target cells and systemic circulation (10, 11).

**Fig. 2:**
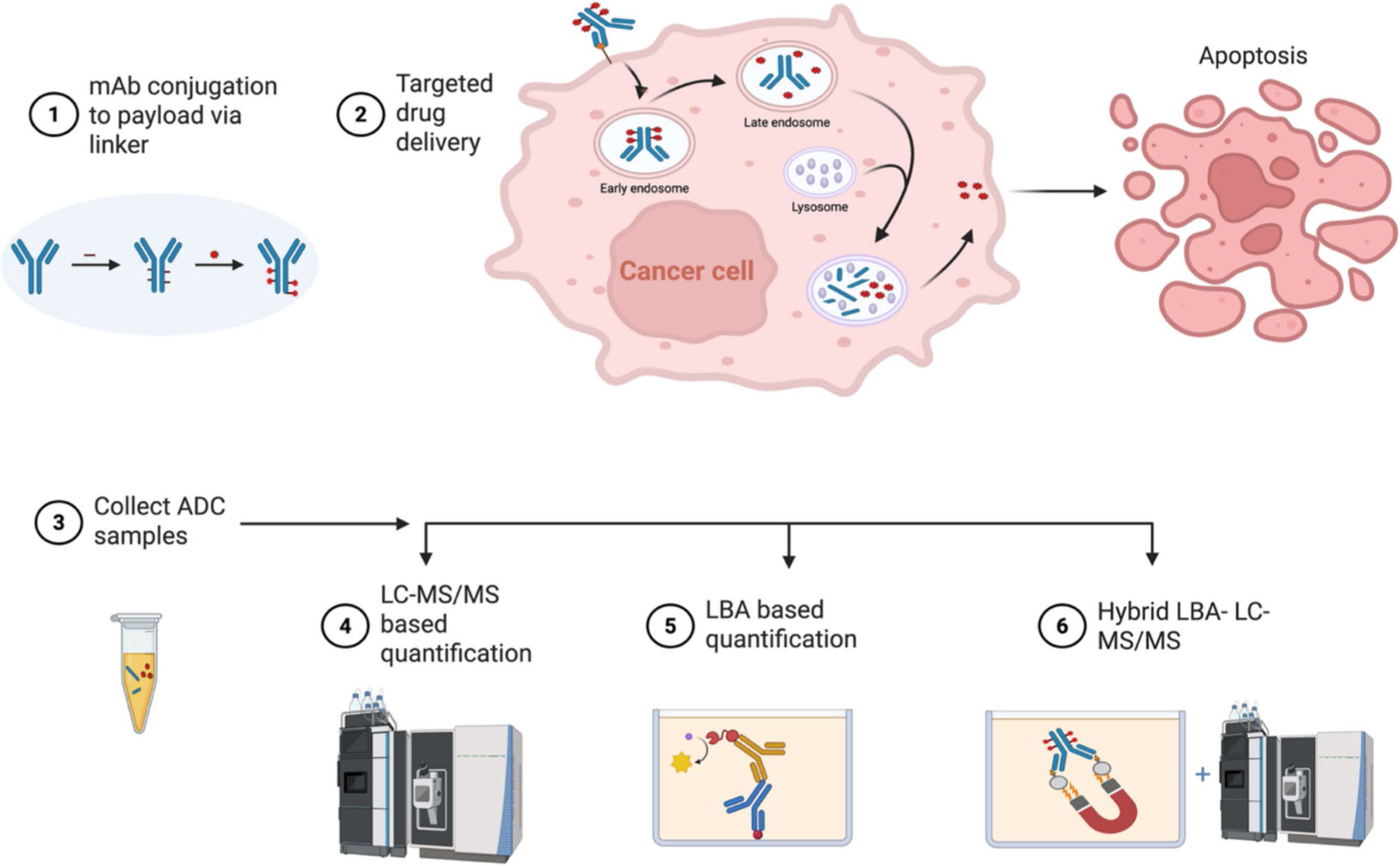
Mechanism of action of an ADC and subsequent quantification by different bioanalytical platforms. **1** mAb is conjugated to cytotoxic payload via a linker to yield an ADC. **2** The ADC binds its target antigen, triggering internalization into the cancer cell. It first enters an early endosome, which then progresses to a late endosome, and if the linker is cleavable, payload cleavage begins here. The late endosome then fuses with the acidic lysosomal compartment to complete enzymatic payload release, resulting in apoptosis. **3** Samples are collected from either preclinical or clinical studies at different time intervals that include various ADC components, **4** that can be quantified either by LC–MS/MS **5** or through ligand binding assays such as ELISA and/or **6** through hybrid LBA – LC – MS/MS quantification. Adapted in parts from references 4 and recreated using biorender.com

Mechanistically, once the ADC reaches the target cell, its antibody component recognizes and binds to the corresponding cell surface antigen, initiating internalization via endocytosis. The ADC is first enclosed within an early endosome, which subsequently matures into a late endosome. This late endosome then combines with a lysosome, facilitating payload release. Once released, the payload induces apoptosis in the target cancer cell (12, 13). The released payload may diffuse beyond the initial target cell, leading to the destruction of neighboring cells, also known as the bystander effect (see Fig. 2) (14). For ADCs with non-cleavable linkers, the payload remains conjugated to the antibody until proteolytic degradation occurs within the lysosome, resulting in a degraded monoclonal antibody (mAb) moiety and a payload with a positively charged lysine, that is trapped in the target cell (15) Alternately, ADCs with cleavable linkers may undergo enzymatic cleavage within the endosome or lysosome, leading to payload release. The bystander effect is commonly observed in ADCs with cleavable linkers non-polar payloads. Payloads with non-polar properties are more membrane permeable, enabling their diffusion into neighboring cells (15). In the context of a heterogeneous tumor microenvironment, where both antigen-positive and antigen-negative cells contribute to cancer progression, the bystander effect theoretically has the potential to improve both therapeutic efficacy and patient outcomes. However, conversely, it can also affect nearby healthy cells (16).

ADCs exhibit a complex PK profile, driven by the distinct properties and interactions of their components, which influence absorption, distribution, metabolism, and elimination (ADME). After the infusion of an ADC, systemic circulation may contain the fully intact ADC or its individual components, including the unconjugated antibody and unconjugated cytotoxic payload. As the large-molecule antibody component displays different PK profiles from the small-molecule payload, PK analysis of an ADC requires a comprehensive understanding of each component. ADC PK is primarily driven by the antibody component, and can display both the benefits and limitations of unconjugated monoclonal antibodies, such as, low volume of distribution, slow clearance, a long half-life, and possibly nonlinear distribution and elimination (17). However, unlike unconjugated antibodies, ADCs, particularly those with higher DAR, tend to exhibit faster clearance due to the addition of the cytotoxic payload (17).

In addition to the inherent complexity of assessing the PK profile of ADCs, accurately characterizing these molecules through bioanalytical analysis presents unique challenges. In contrast to small molecule compounds, which can be reliably quantified using established techniques such as liquid chromatography-mass spectrometry (LC–MS), large biomolecules like ADCs require more sophisticated analytical approaches. This complexity arises from the heterogeneous composition of ADCs, which can exist as fully conjugated, partially conjugated, or completely unconjugated species, each with distinct structural and functional properties that influence safety and efficacy profiles. Therefore, a thorough understanding of ADC PK and pharmacodynamics (PD) requires bioanalytical methods capable of differentiating between fully intact and partially unconjugated forms to accurately characterize their distribution, metabolism, and clearance in biological systems.

There are multiple FDA guidance available on approaches for ADC drug development including bioanalytical assay development and validation and PK considerations (18, 19), as presented below. These methods are extensively used in the pre-clinical phase of ADC drug development as well as in various phases of clinical trials to assess safety and efficacy of the drug.

## Ligand Binding Assays

Ligand binding assays (LBAs) have been widely used for the quantification of large molecules, including ADCs (20). In ADC analysis, LBA’s are primarily used to quantify the total antibody and conjugated antibody components (21), and very rarely, to measure the unconjugated payload (22). LBAs leverage specific antigen–antibody interactions to measure analytes, offering high sensitivity, cost-effectiveness, suitability for high-throughput analyses, and ease of method implementation (Table II) (23, 24). However, LBAs have limitations for ADC characterization, including the inability to holistically differentiate DAR species due to ADC heterogeneity, and challenges in detecting unconjugated payload or partially degraded ADC species. Additionally, the development of specific reagents used in LBAs, such as anti-idiotype antibodies and anti-payload antibodies, can be resource-intensive and time-consuming (24).

## ADC Quantification on LBA platforms

Various types of LBA platforms are currently utilized for ADC quantification, each offering distinct benefits in sensitivity, throughput, and multiplexing potential, with selection of the platform based on the specific requirements of the assay. Commonly used LBA platforms for biotherapeutic analysis include chromogenic, luminescence, time-resolved fluorescence, electrochemiluminescence, label-free, fluorescence polarization, and imaging in combination with existing platforms (30).

For ADC quantification, Enzyme-linked immunosorbent assay (ELISA), Electrochemiluminescence immunoassay (ECLIA), and Gyrolab^®^ have been commonly reported in the literature. ELISA is the most established of the various LBA platforms for PK profiling and immunogenicity testing of large molecules in complex matrices. This is due to its cost-effectiveness and simplicity of procedure (no sample extraction) (24). ELISA is a heterogeneous enzyme immunoassay (EIA) technique in which one of the reaction components is either nonspecifically adsorbed or covalently bound to a solid-phase surface, such as a microtiter well, magnetic particle, or plastic bead, and operates based on the principle of enzymatic signal detection (31). Pie *et al*. (2022) developed an ELISA-based bioanalytical method to quantify Monomethyl auristatin E (MMAE) ADCs and total antibodies in cynomolgus monkey sera. For MMAE-ADC detection, a mouse anti-MMAE antibody served as the capture antibody, while an horseradish peroxidase (HRP)-conjugated mouse anti-human immunoglobulin G – fragment crystallizable (IgG-Fc) antibody was used for detection. To measure total antibody (hRS7), a recombinant human trophoblast cell surface antigen two extracellular domain (hTROP-2-ECD-His) was employed as the capture reagent, with the same HRP-conjugated mouse anti-human IgG-Fc antibody for detection. The assay demonstrated a dynamic range of 0.3–35.0 ng/mL for MMAE-ADCs and 0.2–22.0 ng/mL for total antibodies (32). This underscores the highly sensitive nature of ELISA, particularly for conjugated antibody and total antibody quantifications. Despite the several benefits of ELISA, its use is limited by the potential for matrix interference and limited dynamic range.

ECLIA, like ELISA, is based on the specific interaction between an antibody and its corresponding antigen. It utilizes specialized reagents, including a capture antibody that targets the antigen, and a labeled antibody for detection. To enable detection, the capture antibody is immobilized on a solid matrix, such as a microplate or magnetic bead, while the labeled antibody is tagged with a luminescent marker and an electrochemically active molecule, allowing for signal generation and detection (31). The assay formats used to detect analytes are similar to ELISA: direct, indirect, competitive, and sandwich (33–35). The ECLIA platform is increasingly used to replace traditional ELISA due to higher sensitivity in the femtomolar/10^6^ nM range, wider dynamic range, and minimal matrix effects, as well as limited cross-reactivity with reagents and analytes (36). Skidmore *et al*. (2020) employed an ECLIA-based bioanalytical method to quantify ARX788 (conjugated ADC) and total antibodies in a preclinical study. ARX788 is a site-specific Anti-HER2 ADC generated by conjugating Amberstatin antibody with monomethyl auristatin F (MMAF) payload via a noncleavable hydroxylamine- polyethylene glycol linker. To quantify the conjugated ADC, the anti-Amberstatin antibody (AMB-20) was used as the capture antibody, while biotinylated AMB-20 and streptavidin SULFO-TAG served as detection reagents. For total antibody quantification, a recombinant human ErbB2/HER2-Fc Chimera Protein served as the capture antigen, with detection achieved using a biotinylated goat anti-human kappa light chain antibody and streptavidin SULFO-TAG (37). Nevertheless, a major limitation of ECLIA is the requirement for specialized equipment, reagents, and solvents, making it a more expensive option compared to ELISA (37, 38).

The Gyrolab^®^ system is an advanced automated immunoassay platform that operates within a compact microfluidic disc with preloaded streptavidin columns to facilitate affinity interactions. With the aid of capillary and centrifugal forces, this nanoliter-scale flow-through technology controls liquid flow through the affinity column. By integrating operations within the microfluidic device, the instrument minimizes hands-on time for sample and reagent preparation while maintaining flexibility to accommodate various reagents and analytes. The system is designed to accommodate a wide concentration range and various sample matrices, making it suitable for applications such as biomarker analysis, PK, PD studies, and bioprocess development (39). In a Gyrolab system, the capture and detection reagents may consist of antibodies, proteins, peptides, or nucleic acid-based binders, which can be commercially sourced or developed in-house. Essentially, capture reagents must be biotin-labeled, while detection reagents require a fluorescence tag, such as Alexa Fluor^®^ 647, compatible with the system’s 635-nm red laser detection. Notably, the sample volume used in a Gyrolab^®^ is minimal compared to conventional immunoassays, ranging from 0.02 to 4 *µ* L (40). Okamoto *et al*. (2020) utilized a Gyrolab immunoassay to determine concentrations of trastuzumab deruxtecan (T-DXD) and total antibody in the plasma of HER2-positive tumor-bearing mice and achieved a lower quantification limit of 200 ng/ml (41).

Other advanced LBA immunoassay systems include the Single Molecule Array (SIMOA^™^), a digital ELISA technology by Quanterix, Single Molecule Counting (SMC^™^) from Singulex Inc., Cytometric Bead Array (CBA, Becton, Dickinson and Company), Ella (ProteinSimple/Bio-Techne), and Immuno-PCR (Imperacer^®^) from Chimera Biotec GmbH. These instruments facilitate ultrasensitive biotherapeutic quantification and expedite analytical turnaround times. To the best of our knowledge, these products have yet to be applied specifically to ADC quantification, but their use for antibody quantification has been reported (42, 43), indicating their potential application for ADC bioanalysis.

## LC‑MS/MS Developments in the Quantification of ADCs

**Fig. 3:**
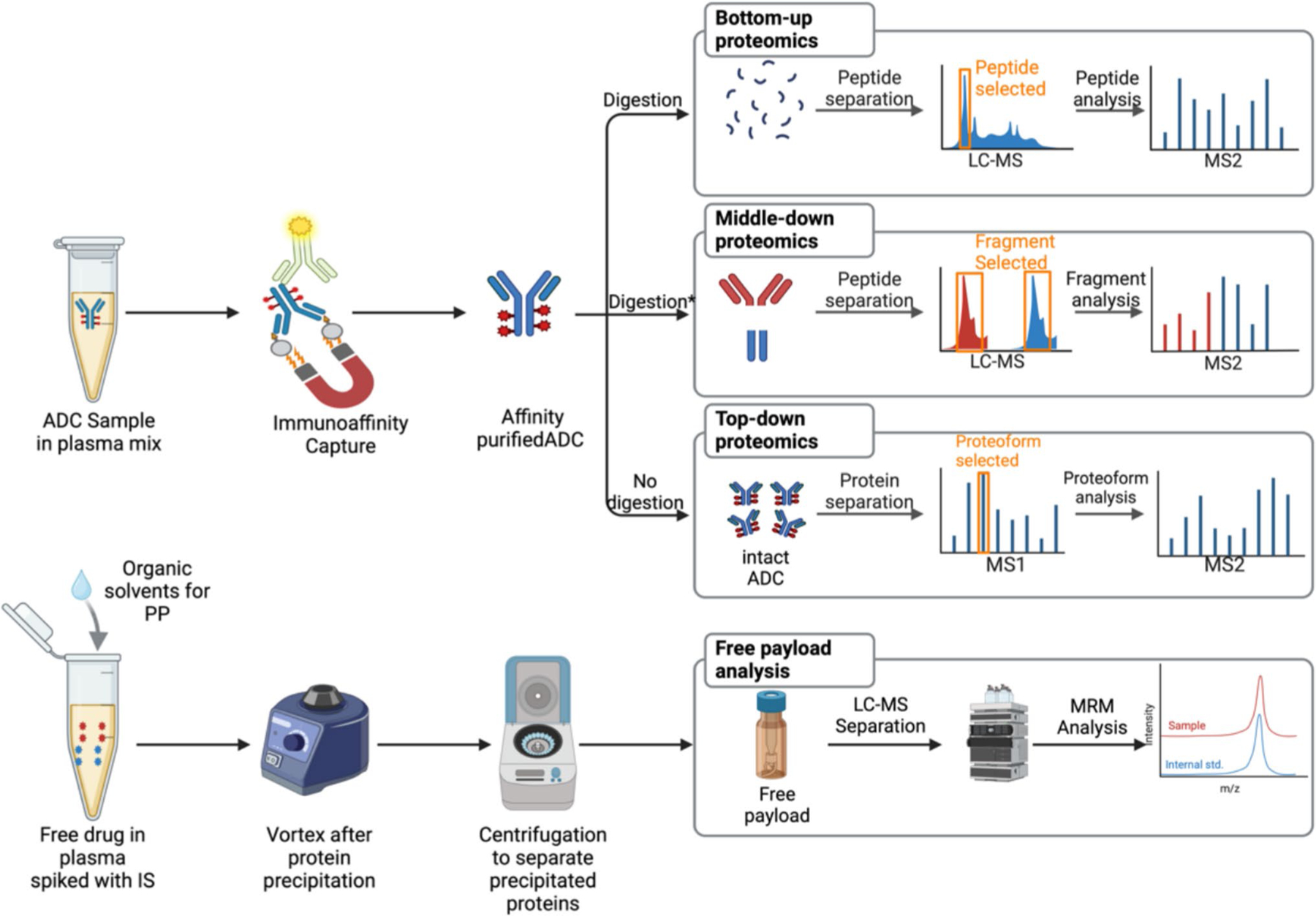
Overview of general sample preparation and LC–MS/MS analysis of ADC and unconjugated payload. ADC from biological matrix is typically enriched using immunoaffinity capture. In bottom-up proteomics, the enriched ADC is digested using trypsin, generating peptide fragments that are then quantified by LC–MS/MS using a surrogate peptide. Middle-down proteomics involves either reducing cysteine-conjugated ADCs with DTT or partially digesting them to separate the light and heavy chain fragments for subsequent LC–MS/MS quantification. The top-down proteomics approach enables direct quantification of the intact ADC. For released payload quantification, the biological matrix is spiked with an internal standard, and plasma proteins are precipitated using organic solvents prior to LC–MS/MS quantification. Adapted in parts from reference 48 and recreated using biorender.com

PK analyses of ADCs have traditionally relied on LBAs, but due to past limitations with LBAs, such as cross-reactivity, limited ability to quantify unconjugated payload, and challenges in differentiating various ADC species, LC–MS-based methods have increasingly been used to improve accuracy and specificity (44, 45). LC–MS methods enable the quantification of ADCs at various levels of structural resolution, including intact analysis (top-down, under native or denaturing conditions) (46), partially reduced analysis (middle-down) (47), and peptide-level characterization (bottom-up) (see Fig. 3) (48). This flexibility allows for a more precise assessment of ADC heterogeneity, metabolism, and clearance compared to traditional LBAs. In addition, this approach allows for sensitive and accurate quantification of the small molecule payload, a critical parameter for assessing both therapeutic efficacy and potential off-target toxicity.

Recently, the LC–MS approach has undergone significant technological advancements. Notably, there have been improvements in ionization techniques, particularly with the development of nano-electrospray ionization, which employs ultra-fine capillary needles to generate highly charged droplets from minimal sample volumes, thereby significantly enhancing analytical sensitivity and resolution (49, 50). Furthermore, there has been an increase in the use of novel mass analyzers, such as the Orbitrap mass spectrometer and the Fourier transform ion cyclotron resonance (FT-ICR) mass spectrometer, which offers exceptionally high mass resolution and accuracy (51, 52). Building on these advances, recent studies have demonstrated the growing use of LC–MS/MS approaches for the quantification of ADCs in biological matrices. A few illustrative examples are highlighted below.

Top-down proteomics involves the direct analysis of intact proteins without digestion, enabling the characterization of sequence variants, post-translational modifications (PTMs), and DARs (Table II). Li *et al*. recently reported an intact quantification workflow for cysteine-conjugated ADCs using native mass spectrometry (nMS) (28), connecting capillary size exclusion chromatography (SEC) directly to the mass spectrometer. Their workflow involved automated affinity purification followed by nMS analysis, allowing monitoring of average DAR and *in vivo* stability of new linker payloads with minimal method development. Mouse PK study samples with two ADCs were analyzed with this method and showed an excellent linear dynamic range of 5–100 μg/mL, and strong linear correlations similar to conventional targeted bottom-up proteomics approach using a surrogate peptide method that had sensitivity from 0.026–100 μg/mL (28).

Deslignière *et al*. also investigated the intact quantification of T-DXd as a model compound to illustrate the potentials of state-of-the-art LC methods coupled to nMS under denaturing conditions using LC–MS and included peptide mapping and middle-down analysis for comparison (53). The samples in this study were either dissolved in water or phosphate buffer with no additional purification employed prior to the analysis. Using nMS, they characterized the DAR as 8, consistent with homogenous cysteine conjugation. The ADC was further digested with IdeS to identify conjugation sites, revealing three major species: a drug-conjugated light chain, a heavy chain (Fd subunit) conjugated to three drugs, and unconjugated Fc/2 regions. Subsequent peptide mapping of T-DXd with trypsin or pepsin digestion provided extensive sequence coverage (96% for heavy chain, 100% for light chain), confirming cysteine conjugations at positions C223, C229, and C232 (heavy chain) and C214 (light chain) (53). Despite growing interest in top-down proteomics, lower sensitivity still remains a challenge, particularly for proteins with very high molecular weight.

Middle-down proteomics bridges the gap between top-down and bottom-up proteomics, providing complementary structural insights. This approach involves partial digestion of mAbs into larger peptides (> 3 kDa), enabling detailed analysis of the primary sequence and accurate localization of drug conjugation sites with enhanced sensitivity. A recent study developed an online LC–MS/MS method coupled with high-resolution Fourier transform ion cyclotron resonance (FTICR) for rapid analysis of reduced ADCs at the subunit level. A concurrent reduction strategy was applied to a model cysteine-linked ADC to generate six distinct subunits: unconjugated light chain (Lc0), drug-conjugated light chain (Lc1), unconjugated heavy chain (Hc0), and heavy chains conjugated with one to three drugs (Hc1–3). This approach enabled simultaneous assessment of partially and fully reduced ADC forms, facilitating rapid and comprehensive characterization of critical ADC attributes, including DAR quantification, primary sequence characterization, and localization of drug conjugation regions (Table II) (26). Yuan *et al*. recently reported a digestion-free middle-down MS (DF-MDMS) approach for quantifying conjugated payloads from four ADCs with varying DARs and linker cleavage properties, using the Zeno TOF 7600 (SCIEX) with collision-induced dissociation (CID) (27). The method employed immunocapture of the ADC using magnetic beads and optional reduction and deglycosylation, enabling sensitive quantification of the conjugated payload (as low as 50 ng/mL) in ADCs with homogenous conjugation. However, it is pertinent to note that multiple factors could contribute to DF-MDMS assay sensitivity. In this study both ADCs with homogeneous cysteine conjugation and with DAR8 showed excellent sensitivity but ADC2 DAR4 and trastuzumab emtansine had much higher LLOQ, even though ADC2 has the same payload as ADC1. Hence, ADCs with stochastic heterogeneous conjugation inherently have a distribution of DAR species, thus affecting the sensitivity of each species (27). In addition, Linker-payload intrinsic ionization and dissociation properties are also key contributing factors towards assay sensitivity of ADCs (27).

Finally, perhaps the most commonly used method, bottom-up proteomics, involves breaking down antibodies into small peptides using proteases, enabling precise site-specific analysis of drug conjugation (Table II) (25, 54). While this approach provides detailed information on conjugation sites, it inherently disrupts the overall protein structure, limiting insights into higher-order architecture. Despite these limitations, bottom-up proteomics remains a valuable tool for characterizing drug-loading patterns or even detailed structural characterization of ADCs (55, 56). Recent studies have further illustrated the utility of bottom-up proteomics for analyzing *in vivo* ADC samples. For instance, a study employing tryptic digestion combined with ligand-binding affinity and LC-MRM^HR^ (multiple reaction monitoring with high-resolution MS) successfully characterized biotransformation products of AZD8205. Using CID (collision-induced dissociation) and EAD (electron-activated dissociation), the study accurately identified payload deconjugation sites, distinguished linker-payload species, and characterized disulfide rearrangements and thiol-adduct formations. Notably, EAD generated diagnostic ions enabling clear differentiation of constitutional isomers and provided structural details of biotransformation species by selectively cleaving disulfide bonds without disrupting the peptide backbone. Thus, demonstrating that bottom-up approaches can effectively quantify and characterize ADC species and DAR variants in complex *in vivo* biological samples (56).

In addition, to assess the drug safety and efficacy of an ADC, it is imperative to fully characterize the released cytotoxic payload in samples after intravenous injection of an ADC. Due to its high sensitivity, LC–MS/MS enables the quantification of released payloads even at very low concentrations. A sensitive and rapid LC–MS/MS method was recently developed to quantify the unconjugated payload, Aur0101, from the investigational ADC PYX-201 in patients with advanced solid tumors (57, 58). Following a simple extraction from 25 μL of human plasma, Aur0101 was quantified by LC–MS/MS with a linear range of 0.036 to 16.823 nM (57). Similarly, Xu *et al*. published a fully validated method to analyze exatecan, its prodrug, and ARV-825 in rat plasma, demonstrating its use in a proof-of-principle PK study of exatecan following intravenous administration to Sprague–Dawley rats (59).

With continued advancements, current research has expanded into incorporating multiple payloads, either two cytotoxic agents or a combination of one cytotoxic payload and one immunoregulating agent into a single ADC. Levengo *et al*. developed a method to construct homogenous dual-drug ADCs (60), while McKertish *et al*. demonstrated synergistic cytotoxic efficacy from dual-drug ADCs (61). Additionally, a recent study has introduced a novel approach that incorporates a cytotoxic payload and an immunoregulating agent into a single mAb for treating triple-negative breast cancer (62). LC–MS/MS remains crucial for quantifying these multi-payload ADCs. Mak *et al*. developed a simple and sensitive workflow enabling simultaneous quantification of six ADC payloads—7-ethyl-10-hydroxycamptothecin (SN-38), Methotrexate (MTX), Deruxtecan (DXd), MMAE (47), MMAF and Calicheamicin (CM) by LC–MS/MS with validated quantification range of 0.4–100 nM for SN-38, MTX, and DXd, 0.04–100 nM for MMAE and MMAF, and 0.4 −1000 nM for CM (63). Using this workflow, the PK profile of unconjugated MMAE was assessed in mouse serum post intravenous ADC administration (5 mg/kg), demonstrating minimal levels of unconjugated MMAE (Cmax = 2.1 ± 0.5 nM) in circulation, thus confirming strong linker stability (64).

Moreover, LC–MS/MS approaches have emerged as a highly efficient and versatile tool for the analysis of ADCs in recent years. Its ability to analyze multiple components of ADCs from a single sample makes the technique highly sought after compared to other available techniques.

## Hybrid LBA‑LC‑MS/MS Assays for the Quantification and PK Analysis of ADCs

Hybrid immunocapture-liquid chromatography coupled with tandem mass spectrometry (LBA-LC–MS/MS) has emerged as a powerful analytical technique for the quantification of ADCs and total antibodies in biological matrices. These hybrid approaches combine the specificity and selectivity of ligand-binding assays with the accuracy, sensitivity, and structural resolution of mass spectrometry, effectively addressing common limitations associated with traditional LBAs (Table II).

Huang *et al*. (29) recently developed a hybrid immunocapture-liquid chromatography coupled with multiple reaction monitoring (LBA-LC-MRM) method for quantifying unconjugated mAbs, site-specific cysteine conjugated ADCs (DAR 2), and site-non-specific cysteine-conjugated ADCs (DAR 8), achieving a linear dynamic range between 1–10 μg/mL. Intact ADC quantification was compared with a surrogate peptide analysis approach targeting peptides from both the light and heavy chains, as well as conjugated payloads. When applied to PK analysis in rat plasma samples, the intact quantification method revealed biotransformation products that were not detected using the surrogate peptide method, demonstrating the additional insights attainable through intact mass spectrometric analyses (29) (Table II).

Similarly, Suh *et al*. evaluated a hybrid micro-flow liquid chromatography-tandem mass spectrometry (μLC-MS/MS) approach for preclinical PK analysis of ADCs using minimal plasma volumes. Their approach utilized semi-automated solid-phase extraction (SPE) to streamline sample preparation and significantly reduce reagent and plasma sample consumption. Compared to traditional LBAs, the μLC-MS/MS platform provided reliable quantification of total antibody, intact antibody, and total ADC in mouse plasma, demonstrating its reliability for robust ADC PK analyses (65).

Further highlighting the versatility of hybrid methods, Yin *et al*. introduced an immunoaffinity LC–MS/MS assay using a signature peptide from the complementarity-determining regions (CDRs) as a surrogate for quantifying TAb levels from PYX-201 in human plasma (66). Collectively, these studies show the effectiveness of hybrid LBA-LC–MS/MS platforms in addressing traditional LBA limitations, such as matrix interference and lack of DAR differentiation, while enhancing analytical rigor and depth in ADC quantification. Building on these advances in quantification, recent efforts have turned toward characterizing the pharmacokinetic behavior of ADCs in greater detail.

## Pharmacokinetic Considerations and Approaches

**Fig. 4:**
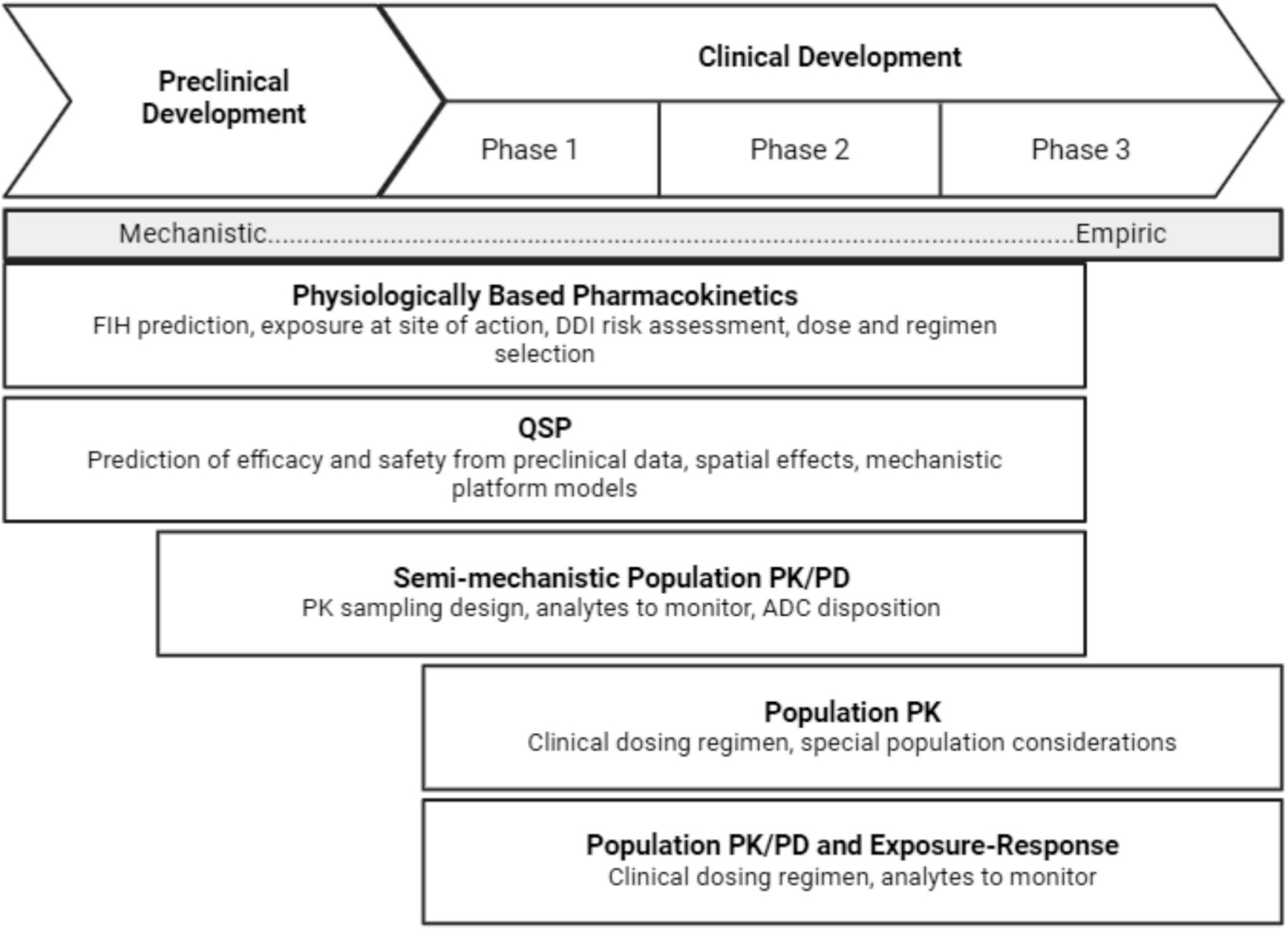
Impact of pharmacokinetic modeling approaches throughout the drug development process. Adapted from reference 66

Characterizing the pharmacokinetics of ADCs can be incredibly complex, as it requires evaluating the PK profile of both the intact conjugate and its individual components – the large molecule antibody and the small-molecule cytotoxic payload – each of which contributes uniquely to the overall disposition and therapeutic effect. Mathematical models are created to characterize the PK characteristics of a drug, i.e., its absorption, distribution, metabolism, and elimination (ADME), and its PD, which includes safety and efficacy endpoints. These models use concentration data obtained from the discussed assays to provide a comprehensive understanding of drug behavior. They are then used to guide decision making, optimize dosing regimens, and predict efficacy or adverse events. Additionally, mechanistic models may be used to simulate drug behavior in on-target and off-target tissues, as well as the deconjugation rate. There are multiple types of models, and their relevance to ADCs is covered further in this section. An overview of modeling approaches across the drug development process can be found in Fig. 4.

Modeling approaches of ADCs must capture the PK/PD of the overall ADC with additional consideration of the variability of the deconjugation process, and the PK of the released cytotoxic payload. Typically, the PK of the ADC is mainly driven by the antibody component. However, the small-molecule cytotoxic payload can be released prior to internalization, diffuse across cell membranes, or affected by efflux transporters, allowing these drugs to escape the target tumor cells and affect nearby cancerous cells or off-target healthy tissues, a phenomenon known as the bystander effect. Although preclinical evidence suggests that heterogeneous tumors may benefit from the bystander effect, it remains challenging to measure this effect in clinical settings (15). Although it is difficult to directly measure, the bystander effect and higher DAR of trastuzmab deruxtecan (T-DXd) is theorized to play a role in the differences found in clinical activity in heterogeneous cancers and cancers with lower target antigen compared to another trastuzumab-based ADC (15, 67). However, recent ADCs with improved linker stability, which would theoretically prevent the induction of the bystander effect, have been shown to be effective even in the absence of a bystander effect, suggesting the bystander effect should not be the sole focus in the development of novel ADCs. (15) Additionally, once the ADC is degraded, the payload can accumulate in certain off-target organs, such as the liver (17) Thus, the appropriate PK modeling approach depends on the specific questions being investigated and the stage of drug development.

Due to the complex nature of ADCs, multiple analytes are collected throughout preclinical and clinical development. During the early clinical phase, the population PK/PD approach is categorized into single-analyte, two-analyte, and semi-mechanistic multiple-analyte models. Single-analyte population PK models focus on the specific analyte determined to be the most clinically relevant to efficacy and safety, primarily the conjugate form, measured as conjugated antibody or conjugated payload. Conjugated antibody concentration is the most commonly used analyte for ADC PK analyses; however, the interpretation of these measurements is complicated by ADC heterogeneity. ADC species with different DARs can exhibit distinct pharmacologic properties, meaning that measured conjugated antibody levels might not reliably predict therapeutic response. On the other hand, assays that quantify the conjugated payload provide limited insight into overall antibody exposure, as low concentrations of ADCs with high DAR may appear similar to high concentrations of ADCs with lower DAR (17). This can complicate the comparison of the PK of an ADC to the expected pharmacologic effect. Improvements in both the analytical methods to better describe the DAR distribution and chemistry techniques to create homogenous ADCs have helped address this issue.

Two-analyte population PK models are far more varied, involving combinations such as total antibody/conjugated payload, total antibody/conjugated antibody, conjugated payload/unconjugated payload, and conjugated antibody/unconjugated payload (68). The two-analyte approach further quantifies the relationship between large-molecule antibody components and the rate of payload deconjugation. It can also model the dynamics of conjugate stability and the subsequent formation and clearance of the unconjugated payload. These population PK approaches also allow for the exploration of covariates or the impact of patient demographics such as body weight, age, sex, ethnicity, and hepatic or renal impairment on the PK. The subsequent population PK-derived exposure metrics, considered better than observed exposure metrics, which are affected by variability across trials and sampling time, are then used for PK/PD modeling or exposure–response analysis and are critical for phase 2/3 dose selection (68). Both further explore the relationship between ADC concentration and efficacy and safety endpoints.

Comparatively, semi-mechanistic models often include multiple-analyte analysis to characterize the mechanism related to proteolytic degradation, deconjugation, and unconjugated payload formation, often built on preclinical data (68). These established models can then be used to predict one analyte from another, decreasing the amount of sampling and bioanalytical assays needed. This semi-mechanistic approach is a blend of the traditional top-down empirical PK/PD approach and the bottom-up mechanistic systems biology approach, resulting in the formation of a new discipline. In recent years, advancements in mechanistic approaches, such as physiologically based pharmacokinetic (PBPK) and quantitative systems pharmacology (QSP) modeling, have enabled further characterization of ADCs. Although QSP is a relatively new discipline compared to PBPK modeling and lacks a universally agreed-upon definition, it has been suggested that PBPK modeling may be considered a subset within the broader scope of QSP. QSP was first defined in a National Institutes of Health (NIH) working group white paper, which recognized the need for a new approach to translating preclinical discoveries into clinical progress by integrating computational modeling of biological and pharmacologic systems (69, 70).

Both approaches require extensive data from multiple analytes obtained through robust assays and often incorporate information obtained from both preclinical and clinical studies. As the name implies, PBPK further integrates physiological information, including different organs and tissues, allowing the estimation of drug concentrations in tissue that would be otherwise difficult to measure in the real world (71). Additionally, PBPK models can be further used to explore possible drug-drug interactions (DDI) of the unconjugated payload and possibly justify approval without a dedicated DDI clinical trial (68). While the complexity of PBPK models have been a barrier, Simcyp Simulator, a PBPK modeling and simulation software, has also released an ADC module that could make further PBPK approaches more accessible. This module was tested using a model for enfortumab vedotin and unconjugated MMAE to predict drug-drug interactions (DDI), demonstrating the applicability of the ADC module during drug development.

There are similarities between QSP and previously discussed methods, as QSP evolved from multiple complementary modeling approaches, including systems biology, PKPD, and PBPK. Although both QSP and PBPK are heavily mechanistic approaches, PBPK models predominantly focus on answering ADME and PK questions, while QSP models focus on PD efficacy and safety outcomes and disease pathophysiology (69). Recent advancements have improved our understanding of intracellular mechanisms, tumor heterogeneity, the bystander effect, and the mechanisms behind ADC toxicity (72). These mechanistic or semi-mechanistic approaches are often favored from preclinical development to the early stages of clinical development, particularly during the transition from preclinical to clinical phases. They are used to leverage preclinical data to predict clinical responses when clinical data is lacking.

**Fig. 5:**
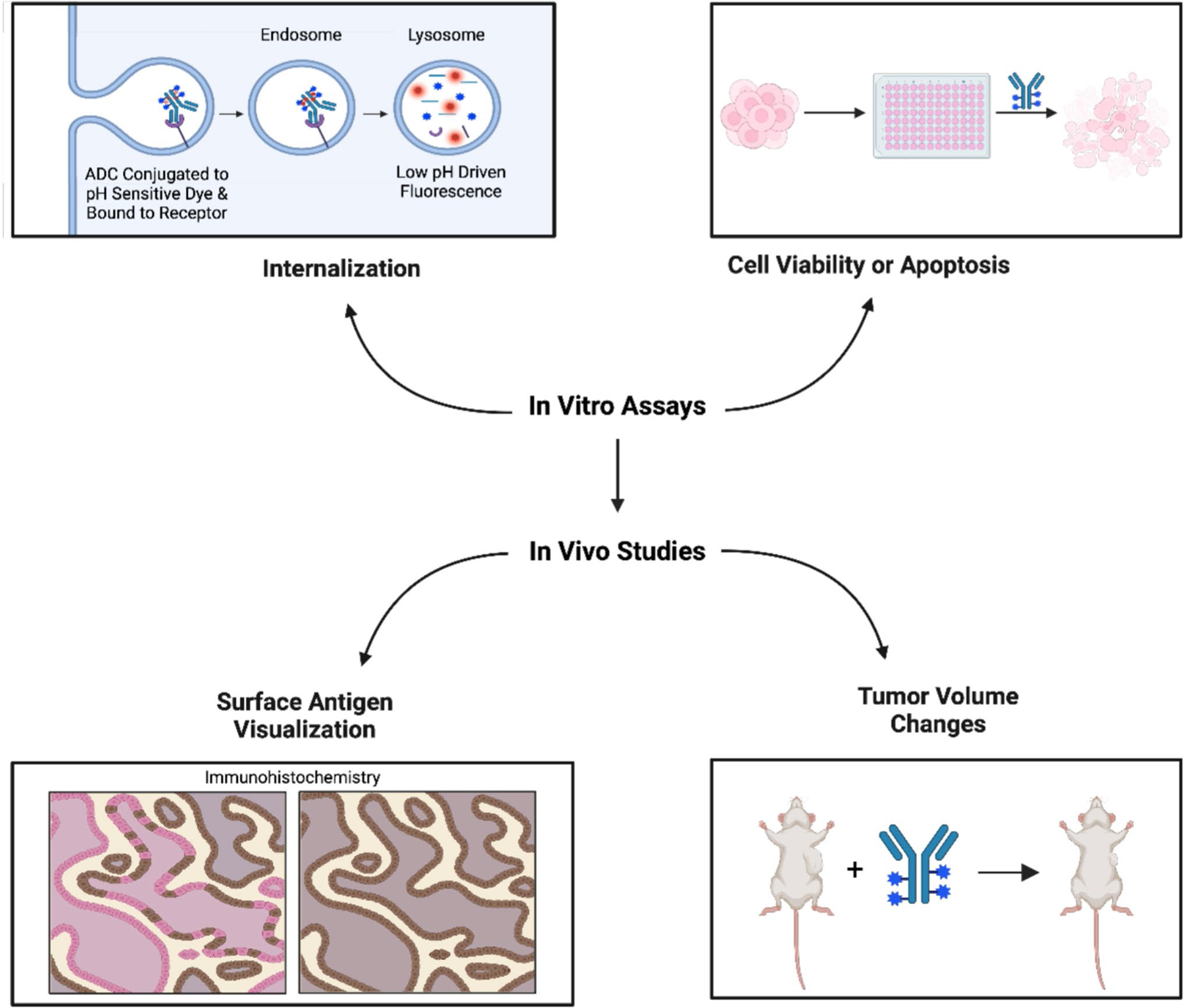
An overview of preclinical techniques to analyze ADC PD *in vitro* and *in vivo*. *In vitro*, pH-sensitive dyes assist in the visualization of the ADC internalization process. ADC-mediated apoptosis or subsequent decreases in cell viability may be assessed in cell-based cancer models. *In vivo*, immunohistochemistry staining of tumor tissue confirms the presence of the cell surface antigen that the ADC targets. In CDXs or PDXs, effective ADCs trigger a measurable decrease in tumor volume

Examples of the preclinical data used depend on the specific aspect of the ADC’s PD being investigated and can be seen in Fig. 5. Internalization of the ADC *in vitro* can be measured by labeling the ADC with a pH-sensitive fluorescent dye (73, 74) and tracking its uptake into an appropriate preclinical model, such as a 2D cell line, 3D spheroid, or organoid (75), using the Incucyte Live-Cell Analysis System or fluorescence microscopy (73, 74). Internalization assays are used to confirm that the ADC enters its target cell enabling payload release. Additionally, by utilizing non-cancerous cell lines, these experiments can reveal whether the ADC enters non-tumor cells, potentially leading to increased toxicity *in vivo*. Once inside the target cancer cell, the preclinical efficacy of the ADC may be assessed using *in vitro* assays that measure apoptosis or changes in cell viability (76, 77). Furthermore, these assays may also be used to evaluate the bystander effect of ADCs *in vitro*. By combining antigen-positive and antigen-negative cells in co-culture systems, and comparing ADC efficacy in monocultures *versus* co-cultures, one can determine whether the ADC facilitates killing of antigen-negative cells through bystander effect mechanisms (78).

Preclinical *in vivo* animal studies also provide valuable data for mechanistic models, as cell line-derived or patient-derived xenografts often offer a more accurate representation of patient drug responses and toxicity compared to *in vitro* systems (79). Prior to treatment, xenograft tumors can be evaluated for antigen expression using immunohistochemistry, with the quantification of antigen levels helping to predict the ADC’s ability to bind target cells (80). ADC efficacy *in vivo* is typically communicated by measuring changes in tumor volume following treatment (73). Additionally, tracking the overall survival of the study animals provides important information on preliminary toxicity, which is crucial for ADCs with a narrow therapeutic index (81). QSP models can then integrate these PD metrics to estimate ADC activity in clinical settings and identify potential off-target effects. Overall, as our understanding of ADCs and bioanalytical methods improves, further advancements in QSP and PBPK modeling can streamline the ADC development pipeline. With further developments in ADC technology, such as improvements in cytotoxic payload conjugation, allowing homogenous high DAR molecules and even multiple payload ADCs, new considerations regarding the PK must be taken into account.

Currently, most ADCs have a single unique cytotoxic payload and exhibit highly variable DAR, which can change over time. The pharmacological activity of ADCs is primarily driven by the cytotoxic payload. However, ADCs with a high DAR tend to have faster clearance than those with a lower DAR, possibly leading to decreased exposure. As a result, most clinical stage ADCs have an average DAR of 3.5–4 (17, 82). Efforts to homogenize ADCs provide more robust and reliable pharmacokinetics, with better plasma stability and fewer off-target effects (83). Currently, Enhertu (fam-trastuzumab deruxtecan-nxki) and Trodelvy (sacituzumab govitecan-hziy), are two FDA-approved ADCs considered to be homogeneous as they approach the theoretical maximum DAR, 8, both of which include cleavable linkers allowing for the bystander effect.

Additionally, there is research into conjugating multiple distinct payloads onto the same antibody. Given the heterogeneous nature of tumors, resistance can develop with many current treatment regimens involving a combination of drugs with different mechanisms of action (84). Theoretically, conjugating multiple payloads onto the same ADC could offer the same benefit and possible synergistic effects as discussed previously. Conversely, there may be concerns about unforeseen toxicity, as there is a current lack of *in vivo* data (85). The pharmacokinetics, safety, efficacy, and possible drug-drug interactions of the individual payloads must also be further considered, as the complex modifications required for multi-payload ADCs may lead to new issues (86). As multi-payload ADCs are still in the early stages of research with limited *in vivo* data, further research and a more comprehensive quantitative approach using the methods mentioned in this review, such as QSP, will be necessary to characterize the safety and efficacy of multi-payload ADCs (86).

## Conclusions

The successful development and clinical implementation of ADC therapeutics are heavily dependent on accurate bioanalytical characterization and subsequent robust pharmacokinetic modeling. This review highlights the importance of analytical methods, including LBAs, LC–MS/MS, and innovative hybrid LCA-MS/MS platforms, in overcoming traditional quantification challenges such as ADC heterogeneity, interference effects, sensitivity, and limited differentiation of analytes. Furthermore, we emphasized the critical role of PK modeling approaches, ranging from population PK methods to mechanistic frameworks like PBPK and QSP, in elucidating ADC disposition, payload dynamics, and drug tissue interactions. The integration of these models enables researchers to better understand and predict ADC behavior, which is crucial for informing dosing strategies and therapeutic decisions. It is evident that future advances in ADC design, including multi-payload integration and optimization of DAR homogeneity, will necessitate further refinement of analytical methods and PK modeling strategies. By combining state-of-the-art bioanalytical quantification and sophisticated PK modeling methods, scientists will be able to gain deeper insights into ADC pharmacology, streamlining the development process and enabling more effective, safer therapeutic options for patients.

## Figures and Tables

**Table I:** FDA-approved ADCs and the Bioanalytical Assays used for the Quantification of ADCs or their Components in Serum or Plasma for Submission

Brand	Generic	Approval Year	Target	Bioanalytical Assay
**Mylotarg** *	gemtuzumab ozogamicin	2000; 2017	CD33	ELISA LC–MS/MS
**Adcetris**	brentuximab vedotin	2011	CD30	ELISA LC–MS/MS
**Kadcyla**	trastuzumab emtansine	2013	HER2	ELISA LC–MS/MS
**Besponsa**	inotuzumab ozogamicin	2017	CD22	ELISA LC–MS/MS
**Polivy**	polatuzumab vedotin	2019	CD79b	Immunoaffinity LC–MS/MS
**Padcev**	enfortumab vedotin	2019	Nectin-4	ELISA LC–MS/MS
**Enhertu**	trastuzumab deruxtecan	2019	HER2	ECLIA LC–MS/MS
**Trodelvy**	sacituzumab govitecan	2020	TROP2	ECLIA LC–MS/MS
Blenrep*	Belantamab mafodotin	2020	BCMA	ECLIA LC–MS/MS
**Zynlonta**	loncastuximab tesirine	2021	CD19	ECLIA LC–MS/MS
**Tivdak**	tisotumab vedotin	2021	TF	ELISA LC–MS/MS
**Elahere**	mirvetuximab soravtansine	2022	FRα	ELISA LC–MS/MS
**Datroway**	datopotamab deruxtecan	2025	TROP2	Gyrolab LBA LC–MS/MS

* Mylotarg was withdrawn in 2010 and reapproved in 2017. Blenrep withdrawn in 2022 but has been reapproved in the UK in 2025

**Table II:** Comparison of Various Analytical Methods Employed for the Quantification of ADCs

Analytical Method	Principle	Advantages	Limitations	Typical Analytes Measured	Quantification Range
Ligand-Binding Assay (LBA) (21–23)	Specific antibody-antigen interaction	- High throughput - Cost-effective - High sensitivity for antibodies	- Cross-reactivity issues - Limited DAR specificity - Matrix interference	- Total antibody - Conjugated antibody - Payload (rarely)	Medium—High
LC–MS/MS (bottom-up) (25)	Proteolytic digestion followed by mass spectrometry	- High specificity - High sensitivity for payloads - Site-specific information	- Time-consuming sample preparation - Loss of intact structural context	- Payload - Site-specific conjugation	Medium—High
LC–MS/MS (middle-down) (26, 27)	Partial digestion/reduction followed by mass spectrometry	- Good balance between structural detail and simplicity - Localization of conjugation sites	- Intermediate complexity of data interpretation	- Payload - DAR - Partially reduced ADC components	Medium
LC–MS/MS (top-down, intact) (28)	Direct analysis of intact ADCs via high-resolution mass spectrometry	- Retains intact structural context - Rapid workflow - Direct DAR measurement	- Lower sensitivity than bottom-up - Complex spectral interpretation	- Intact ADC - DAR distribution	Low—Medium
Hybrid LBA–LC–MS/MS (29)	Immunocapture enrichment combined with LC–MS/MS quantification	- Combines specificity of LBA and accuracy of MS - Overcomes traditional LBA limitations (DAR resolution)	- Method development can be complex - Requires specialized instrumentation	- Total antibody, - Conjugated antibody - Payload	Medium—High
